# The American and Colonial Journals: Pathology

**Published:** 1841-07

**Authors:** 


### II. THE AMERICAN AND COLONIAL JOURNALS.

*Remarks on Dyspepsia as connected with the Mind.* By A. Flint, m.d., Buffalo.

[The view taken of a large class of dyspeptic diseases by the author of this excellent paper, is so accordant with our own observation and experience, and has so long successfully guided our practice, that we gladly transcribe the greater part of it, and earnestly recommend the attentive study of it to the host of drug-doctors in this country, whose name is "Legion."]

A dyspeptic presents himself to a physician and states his sufferings. The physician inquires concerning his habits, mode of life, &c. He ascertains that he has indulged his appetite for food and drink without much discrimination, and at all periods has neglected to take exercise, &c. He exclaims at once, "it is not surprising that you have dyspepsia," and he recommends him to reform his habits. If the patient follows his advice perhaps he recovers his previous health without difficulty. Shall we then say that dyspepsia generally arises from dietetic errors? This, probably, is the common doctrine; but I am disposed to doubt its general applicability. How many are there who pursue such a course for a great length of time, perhaps during the whole period of life, without becoming dyspeptic, when, as far as we can judge, there is no reason to suppose their immunity is owing to a better constitution or stronger power of endurance! On the other hand, how many with the utmost care and prudence become and continue dyspeptics! It is not to be inferred that irregularities and intemperance are not common causes of this disease. In the poorer classes, it may, perhaps, generally be attributed to these, conjoined with destitution, bad food, sedentary occupations, close or pernicious atmosphere, exposure, &c.; in the rich to the abuse of luxuries, with love of ease. But there is a class who do not want the comforts of life, and who do not indulge in luxurious excesses, and observation shows, that in this class dyspepsia is not only common, but peculiarly obstinate and persistent. This is an important fact in relation to the disease. A poor person, if he be better fed and clothed, his habits and the circumstances about him improved, is restored. A rich man, if he curtails his pleasures, uses more exercise, engages in healthful occupation, may, in general, expect the speedy return of good health and spirits. But the class between these extremes, who are already clothed and fed, who have no excesses to curtail, find, whatever course they may pursue, that to overcome their difficulties, requires not a small degree of care and perseverance. Physical peculiarities of constitution may explain this in some cases, but, in general, it seems to me, that the explanation is to be derived from the connexion of the disease with the mind. This class, it is to be observed, as a general remark, embraces that portion of mankind, who are disposed more or less to occupations or pursuits which involve, in a greater degree than in the other classes, the exercise of the intellect. Dyspepsia, it has been always observed, is more liable to seize upon those who are thus disposed, and two reasons have been assigned for this preference; viz. 1. The sedentary habits which these pursuits and occupations generally involve. 2. Reasoning from the well-known sympathy which exists between the brain and stomach, to excessive or disproportionate cerebral exercise. There cannot exist a doubt that the former play an important part in the production of, and predisposition to, the disease, but with regard to the latter, so far as I am able to judge from my own observations, it is rarely a cause of the disease, excepting in as far as it involves the former. Among literary men we do not find that they are so liable to the disease who are in the habit of intense, prolonged, or frequently repeated intellectual exertions, although accompanied with much excitement and perturbation, as those whose exertions are of a plodding character; and these generally seem to suffer in the way as some artisans, viz., from the deficiency of muscular exercise, the invigoration of the atmosphere, &c. On the other hand, deficiency of intellectual exertion as a cause of the disease, seems to have escaped observation. I have been led to think that this in certain mental constitutions peculiarly predisposes to dyspepsia. "Mind tends to action," or, to quote the expression of another physiologico-philosopher, exercise or action is a "want" of the intellect. This tendency or want will exist in proportion to the extent of the mind's capacity for exertion, and, like all the instinctive impulses and demands implanted in the human constitution, it must be fulfilled and gratified, or the economy will suffer. If we carefully examine the history of the cases which fall under our observation, we shall find that a large number of them, although in their details or particulars they may differ, are, nevertheless, to be associated, as it regards the causes which have produced and which perpetuate them under this common principle. Nor is it intended to apply these remarks exclusively to those who are pre-eminently intellectual persons. The mental energy may be expended on other than literary and scientific objects, in the performance of any occupation not wholly mechanical, in the fulfilment of the various responsibilities of life; and its obstruction as it regards the latter may be attended with the same results as in the former case. According to this view, the disease under consideration, is consequent to the unnatural condition in which many individuals are placed as it regards the exercise of the various faculties and powers of mind; or, in other words, to a want of correspondence between the mental constitution and extrinsic circumstances. By the term mind, and the expression mental constitution, I would embrace all that appertains to the moral as well as intellectual powers and faculties. My remarks have had more particular reference to the latter, but in many, if not the larger proportion of individuals, the wants of the moral nature, the affections and sentiments, predominate over those which are purely of the intellect, and there is reason to believe that similar results may follow their obstruction or perversions. Indeed, it is probable that instances of the latter are more common than of the former.

It may be said, on the supposition that this explanation of the origin of the disease be correct, why are not its peculiar aberrations the direct effect of causes operating on the mind, without the intervention of the digestive organs. This is not probable in the first place, from the constancy with which they are associated with derangement more or less of these organs, together with their distinctive traits; and in the second place, it is not presumed that all cases of dyspepsia originate in this manner. The successive agents, then, in the development of the disease will be threefold:—

1. The operation of the mental causes.
2. The affection of the digestive apparatus.
3. The reaction of the latter upon the mind, producing those mental aberrations which characterize the disease.

From this doctrine is derived a sufficient explanation of a fact which has been mentioned, viz., that dietetic errors are persisted in often with impunity by those whose strength of constitution and powers of endurance are apparently no greater than of those who suffer.

*Treatment.*—It is well known, that in numerous cases, all the various modes of medical treatment recommended, accomplish but little toward restoring the patient to a healthful condition. The truth is, in the majority of cases, the patience of the physicians is exhausted, by the inefficacy of the remedies prescribed; or the patient, after application to different members of the profession, and experimenting with the thousand and one empirical nostrums, relinquishes all expectation of benefit from the materia medica. But the inquiry arises, if it be true that the disease, in a large number of cases, is to be attributed to causes existing in the mind of its relations, would not the philanthropic physician be able to afford, in many instances, effectual service by suggesting measures which have reference to these, in addition to those appertaining to the materia medica? It is too common for medical advisers to pay but little regard to the mental aberrations peculiar to this disease, thinking that, in the language of Skakspeare, "Therein the patient must minister unto himself." To examine them with attention, and, if possible, to afford relief, would, under any circumstances, be embraced within that philanthropy which should be inseparable from the practice of our profession; but, since they depend upon physical causes, they are to be regarded as morbid symptoms, and fall, legitimately, within the province of the healing art. If physicians were more generally and fully impressed with this view of the subject, perhaps the disease under consideration would become less an opprobrium than it, confessedly, now is.

It may then be stated, as the first important rule in the treatment of the disease, to ascertain fully the kind and degree of mental aberration which exists. To listen with patience and sympathy to all the changes of feeling which the patient is ready to describe, if he receives encouragement to do so, is, in itself, a source of much consolation, and goes far to secure to the medical adviser the possession of the entire confidence of the sufferer. In connexion with this, the mental characteristics of the individual, his habits, education, &c. are to be considered, both as enabling us better to appreciate the nature of the changes which have occurred, to decide upon the remote causes of the disease, and to determine upon the measures to be recommended with a view to restoration.

In the second place, it is important to satisfy the patient that the altered condition of his mind and feelings is symptomatic of a morbid condition of body. This is often so little suspected, that his unhappy state is not described until inquiry is made relative to this point. But, as soon as the patient finds it is suspected as associated with the disease, he gladly becomes communicative. It is truly pleasing to witness the surprise and animation which lights up the sombre countenance of the unfortunate dyspeptic, when he finds that the state of his feelings is anticipated by the physician. He seems to hail it as a favorable omen. If the idea has never been suggested that his unhappy condition is the effect of a disordered body, it furnishes the first occasion for hope; and whether restoration is effected or not, he is enabled to resist and sustain his trials with more fortitude and perseverance.

The next object will be to endeavour to remove the causes which have originated or which maintain the disease. But inasmuch as these are very multifarious, and their different varieties have not been considered, the remarks upon therapeutical principles will of course be very general. Each case, in fact, should form a separate study; but in general terms, the patient should be urged to provide that particular kind of stimulus for the intellectual and moral powers, which he seems to require.

In some instances, the difficulty seems to consist chiefly in the monotony incident to routine duties. Then, the indication is to vary their character, or advise a temporary interruption. In such cases, travelling is highly useful; but, frequently, to be permanently efficacious, it should not be confined within a narrow sphere of time or space. Especially foreign travel is useful by the increased excitement and interest derived from the comparison of scenery, and the habits, manners, and institutions of other countries. But this unfortunately is a measure which only in a small number of cases can be adopted. Those means alone can be embraced which are accessible at home. These, however, are not few or powerless. Sometimes it may be proper to advise an entire change of occupation and locality, in order to supplant completely old by new associations. This method, which has been found of such utility in mental derangement, would, probably, be not less so in cases of partial alienation, as these cases must be regarded.

When this is not advisable, or practicable, other measures must be adopted to rouse the faculties of the intellect and the moral sentiments. One of these is the commencement of certain branches of study, or some plan of intellectual effort. Those departments which are pursued by means of observations and experiments rather than abstract contemplations are to be preferred. This will of course apply only to those who have leisure, inclination and capacity for such occupations, and to that class who require more especially excitement of the intellectual faculties.

In other cases, the social and moral sentiments are to be operated upon by the formation of new connexions, assuming new responsibilities, and by directing the mind to objects which are calculated to engage the feelings of benevolence and philanthropy; such are politics, the cause of popular education, and the numerous particular plans of every scale of magnitude, tending to the amelioration and improvement of the human race.

The selection of any of these measures, as has been already remarked, will depend upon the combination of circumstances which distinguish the cases individually, and is to be left to the discrimination of the medical adviser. The hearty co-operation of the patient is of course requisite to the prosecution of any plan, and with a view to this the whole subject should be fully discussed and the state of the case frankly stated. One good result will at least accrue from such a course; it will tend to preserve feelings of respect for the character of the medical profession with a class of patients whose experience of it too often leads them to entertain opposite sentiments.

*American Journal of the Medical Sciences. January,* 1841.

---

### III. THE BRITISH JOURNALS.

(FOR THE QUARTER ENDING MAY 30, 1841.)

